# Metachronous Primary Adenocarcinoma of Distal and Proximal Ureter within Two Years

**DOI:** 10.1155/2014/659258

**Published:** 2014-06-02

**Authors:** Dominik Abt, Gautier Müllhaupt, Livio Mordasini, Pierre André Diener, Hans-Peter Schmid

**Affiliations:** ^1^Department of Urology, Cantonal Hospital St. Gallen, Rorschacherstraße 95, 9007 St. Gallen, Switzerland; ^2^Institute of Pathology, Cantonal Hospital St. Gallen, Rorschacherstraße 95, 9007 St. Gallen, Switzerland

## Abstract

Primary adenocarcinoma of the upper urinary tract, particularly of the ureter, is an extremely rare entity. We are reporting on the first case of metachronous appearance in one patient. The 71-year-old man underwent partial ureterectomy (R0 resection) for primary adenocarcinoma of the left distal ureter. 3 years later, nephroureterectomy had to be performed because of metachronous primary adenocarcinoma of the left proximal ureter. Extensive examinations revealed no evidence for further malignancies at both times. Primary adenocarcinoma of the upper urinary tract is rare but should be kept in mind, especially in patients with chronic inflammation and urinary tract obstruction. Due to the low incidence, there is a lack of data regarding its pathogenesis, diagnosis, and optimal treatment.

## 1. Introduction

Malignancies of the upper urinary tract are rare. Approximately, 90% of them are transitional cell carcinomas (TCC); squamous cell carcinomas account for less than 10%. Primary adenocarcinomas of the upper urinary tract are extremely rare, representing less than 1% of all tumors [1]. There are only a small number of reported cases of primary ureteral adenocarcinoma and we are reporting on the first case of metachronous appearance in one patient.

Adenocarcinomas of the urinary tract are subdivided into tubulovillous, mucinous, and papillary nonintestinal categories. Tubulovillous and mucinous tumors represent intestinal adenocarcinomas and constitute 93% of the cases [1, 2].

Mucinous adenocarcinomas are presumed to be originating from intestinal metaplasia of the transitional epithelium and to have a better prognosis, while the papillary nonintestinal variety occurs in younger individuals and is not necessarily associated with inflammation [1].

However, there seems to be a frequent association of upper urinary tract primary adenocarcinoma with chronic irritation, inflammation, infection, hydronephrosis, and urinary calculi. Thus, glandular metaplasia of the urothelium due to injury or inflammation might eventually progress to dysplasia and adenocarcinoma [3, 4]. A noticeable accumulation of cases from Japan and India also supports an inflammatory, environmental, or dietary etiology [5, 6].

## 2. Case Presentation

**Figure 1:**
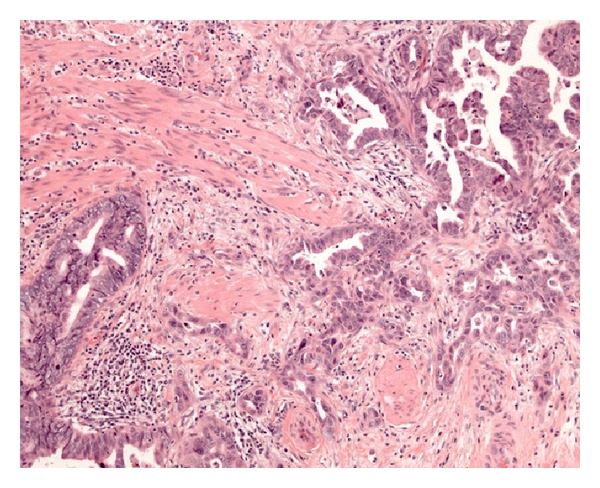
Adenocarcinoma invading muscularis, H and E, 100x.

The 71-year-old was referred to our clinic 3 years ago after an incidental finding of asymptomatic left sided hydronephrosis. None of the above-mentioned risk factors (i.e., inflammation, infection, hydronephrosis, and urinary calculi) could be found in the reported case. Moreover, the patient had no smoking history, neither a family history of cancer. While cystoscopy showed no visible tumor, retrograde ureteropyelography demonstrated ureteral stenosis, 4 cm, proximal to the ureteral orifice, not accessible for biopsy. Selective ureteral cytological evaluation of urine indicated high-grade TCC. Staging was performed by thoracoabdominal computed tomography (CT) and revealed localized disease. As the patient refused radical nephroureterectomy, partial ureterectomy with ureteroneocystostomy was performed. Histology disclosed a 10 × 9 × 7 mm lesion of an adenocarcinoma pT3, pN0 (0/11), G2, R0 (Figure 1). Colonoscopy for colorectal cancer screening two weeks prior to surgery had shown no signs for malignancy or dysplasia.

**Figure 2:**
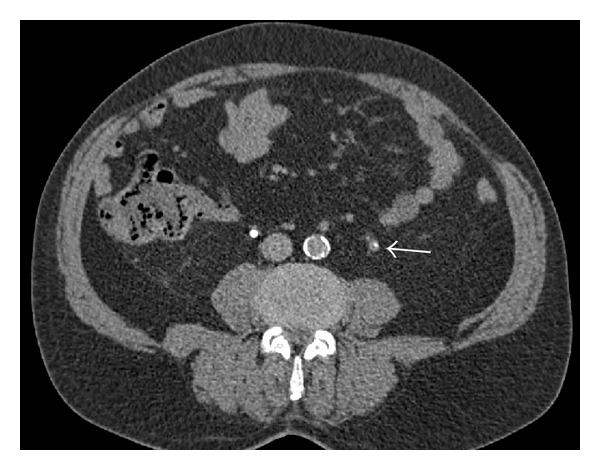
CT scan showing ureteral wall thickening with enhancement of contrast medium.

**Figure 3:**
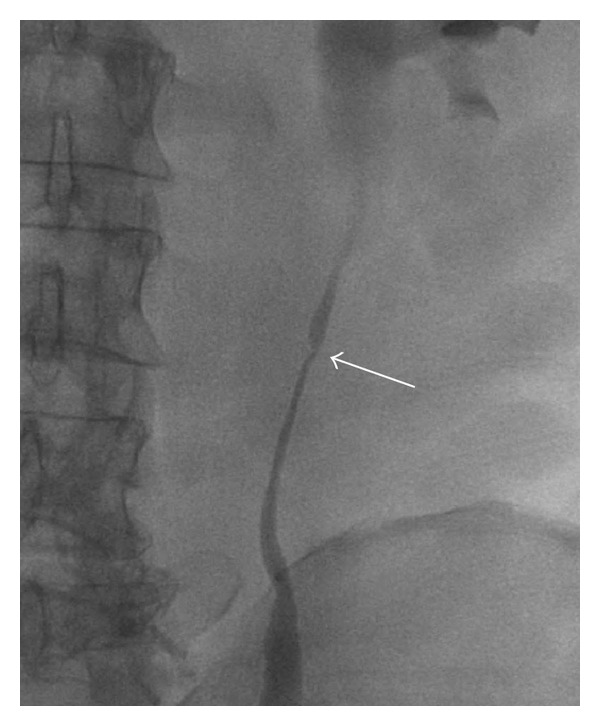
Retrograde ureteropyelography showing ureteral stenosis at the L3 vertebral level.

Followup controls were scheduled on a regular basis (3 and 6 months after operation and every 6 months afterwards) by CT, cystoscopy, and cytology without signs of recurrence. After 2 years, CT scan showed a ureteral wall thickening with enhancement of contrast medium (Figure 2). The patient was free of symptoms, despite a proximal ureteral stenosis at the L3 vertebral level in retrograde ureteropyelography (Figure 3).

**Figure 4:**
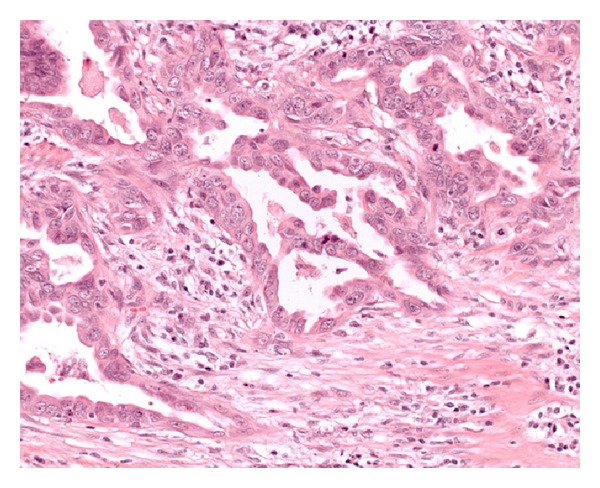
Infiltrating adenocarcinoma G2, H and E, 200x.

As before, the lesion was not accessible for biopsy and cytological examination showed atypical cells interpreted as high grade TCC. This time the patient underwent radical nephroureterectomy for pT3, NX, G2, R0 adenocarcinoma of the left proximal ureter (Figure 4) with absence of tumor or dysplasia in the remaining parts of the ureter. Immunohistochemical examination showed expression of the cytokeratins CK 7, 8, 17, and 20, but no expression of CK 5, 6, and 13. In contrast to CA 19-9, CDX2, and PSA, also expression of CEA and focally thyroid transcription factor 1 could be shown. These findings are consistent with nonmucinous adenocarcinoma of intestinal type.

FDG-PET/CT and colonoscopy showed no evidence for another primary tumor or metastatic disease and elevation of serum CEA could be excluded. At followup, performed by CT, cystoscopy, and cytology after 6 and 12 weeks and after 6 and 12 months, so far, no signs of recurrence occurred.

## 3. Discussion

Primary adenocarcinoma of the upper urinary tract is a rare tumor entity. However, it should be kept in mind, especially in patients with urinary calculi accompanied by chronic inflammation and urinary tract obstruction. In most cases precise diagnosis is not made before surgery, because surgery is often performed for urinary calculi or chronic infection and there is a low concordance of prior cytology subclassification results [7, 8]. While all cases reported were treated by radical nephrectomy or nephroureterectomy, the value of adjuvant chemotherapy and disease prognosis cannot be appraised due to lacking of data. In the reported case the patient did not have any of the above-mentioned risk factors (i.e., chronic irritation due to inflammation, infection, hydronephrosis, urinary calculi, or smoking). Moreover, partial ureterectomy showed R0 resection and the second appearance of adenocarcinoma occurred two years later, located within the proximal ureter with the remaining ureter being free of tumor or dysplasia. As no glandular elements can be found in the upper tract urothelium, pathogenesis of adenocarcinoma must be considered as metaplastic. Thus, like in transitional cell carcinoma, there might be a generalized susceptibility of the whole urothelium to develop metaplasia or adenocarcinoma. This possibility in general, as well as the present case, might indicate that surgery for upper tract adenocarcinoma should be handled according to transitional cell carcinoma. This might also be the case for followup regimens, for which recommendations regarding extend and timing are lacking. As the patient did not have any family history of cancer, genetic testing for syndromes like hereditary nonpolyposis colorectal cancer (HNPCC) was not performed in this case. However, both family history and genetic testing might be helpful to identify patients at high risk for future malignancy.
